# Effects of Noise and Serial Position on Free Recall of Spoken Words and Pupil Dilation during Encoding in Normal-Hearing Adults

**DOI:** 10.3390/brainsci11020277

**Published:** 2021-02-23

**Authors:** Miseung Koo, Jihui Jeon, Hwayoung Moon, Myungwhan Suh, Junho Lee, Seungha Oh, Mookyun Park

**Affiliations:** 1 Department of Otorhinolaryngology-Head and Neck Surgery, Seoul National University Hospital, Seoul 03080, Korea; misng9@gmail.com (M.K.); drmung@daum.net (M.S.); junlee@snu.ac.kr (J.L.); shaoh@snu.ac.kr (S.O.); 2 Yeongeon Medical Campus, Seoul National University College of Medicine, Seoul 03080, Korea; wlgml0720@snu.ac.kr (J.J.); hymun1372@snu.ac.kr (H.M.); 3 Sensory Organ Research Institute, Seoul National University Medical Research Center, Seoul 03087, Korea

**Keywords:** working memory, listening effort, hearing in noise, free recall, pupillometry, cognitive demand, memory, reading span, baseline, serial position

## Abstract

This preliminary study assessed the effects of noise and stimulus presentation order on recall of spoken words and recorded pupil sizes while normal-hearing listeners were trying to encode a series of words for a subsequent recall task. In three listening conditions (stationary noise in Experiment 1; quiet versus four-talker babble in Experiment 2), participants were assigned to remember as many words as possible to recall them in any order after each list of seven sentences. In the two noise conditions, lists of sentences fixed at 65 dB SPL were presented at an easily audible level via a loudspeaker. Reading span (RS) scores were used as a grouping variable, based on a median split. The primacy effect was present apart from the noise interference, and the high-RS group significantly outperformed the low-RS group at free recall measured in the quiet and four-talker babble noise conditions. RS scores were positively correlated with free-recall scores. In both quiet and four-talker babble noise conditions, sentence baselines after correction to the initial stimulus baseline increased significantly with increasing memory load. Larger sentence baselines but smaller peak pupil dilations seemed to be associated with noise interruption. The analysis method of pupil dilation used in this study is likely to provide a more thorough understanding of how listeners respond to a later recall task in comparison with previously used methods. Further studies are needed to confirm the applicability of our method in people with impaired hearing using multiple repetitions to estimate the allocation of relevant cognitive resources.

## 1. Introduction

Working memory capacity (WMC) is defined as the limited but flexible cognitive capacity of an individual to store degraded speech input in memory and process it [1]. WMC reportedly plays an important role in making the temporary storage and manipulation of information more efficient [2,3]. In this regard, a question about cognitive benefits from amplification strategies of hearing aids beyond improved clarity and audibility has been raised [4]. Several studies report that people with high WMC outperform those with low WMC in speech perception tasks [5,6,7]. The question posed above also seems to relate to listening effort, defined as “the deliberate allocation of mental resources to overcome obstacles in goal pursuit when carrying out a task that involves listening” [8]. Significant reduction in listening effort, indicated by pupil dilation, was found in users of hearing aids with a noise-reduction scheme compared to absence of a noise-reduction scheme [9]. Such effort is greatly increased by adverse listening conditions, which are usually associated with increased cognitive demand and reduced performance [10].

Several tests have been developed for indirect measurements of listening effort [11,12,13]. Using sentence-final word identification and recall test (SWIR), Ng et al. [13] found that hearing-impaired listeners performing free recall of spoken words benefited from binary masking noise reduction, and the SWIR tasks, include asking them to repeat the target word immediately after hearing each sentence and to recall target words after each list of sentences. The free-recall task was designed to estimate a listener’s cognitive spare capacity with seven-sentence lists. Using listeners’ reading span (RS) scores [14], a reliable measure of verbal WMC, Ng et al. also found RS scores to have a significant correlation with the results of SWIR in quiet and stationary noise, and a statistically significant association was found between RS scores and recall performance in four-talker babble noise [13]. Lunner et al. [11] found a similar relationship of RS scores with recall performance when the listening condition involved noise-reduction processing. Moreover, recall performance varied significantly with WMC; in both studies [11,13], significant effects of serial position [15] on free-recall performance were present, with improved recall of first and last items compared to the middle items in retrieval of words-to-be-remembered, thereby forming the U-shaped curve of list recall. This conservative method of scoring reflecting the serial position effect has long been used in recall-based assessments; the primacy effect reportedly depends on long-term memory, while the recency effect depends on short-term memory. Another early free-recall study performed on normal-hearing adults by presenting the Revised Speech Perception in Noise Test (R-SPIN; lists of eight sentences) also reported primacy (early in the list) and recency (late in the list) effects [16]. Recall of the last words was relatively consistent compared to the primacy effect, which was variable and was dependent on the signal-to-noise ratio (SNR) of the speech [12]. 

Pupillometry is a rapid physiological measure of cognitive load [17,18]. Recent studies on cognitive hearing have focused on effort-related or task-evoked pupil dilation, and its neurobiological mechanisms are reportedly sensitive to task demands as a result of modulation in arousal linked to locus-coeruleus norepinephrine activity [19]. Pupil dilation refers to an increase in pupil diameter between the onset and offset of a stimulus, and is calculated relative to the baseline pupil size (or baseline) measured during the pre-stimulus (quiet or noise-only) period. Listening effort and its relationship with cognitive abilities have been widely explored in regards to performance in speech-in-noise perception, and, among various parameters, peak pupil dilation (PPD), relative to baseline, is known to reliably reflect varying levels of listening effort, which are affected by adverse listening conditions or the task demand [20]. However, few studies on memory-recall performance of speech where multiple stimuli are presented in each trial have been conducted to examine how these two parameters, baseline and PPD, are associated with each other and to report which method is optimal for the memory-recall data set. A study of speech-recognition found elevated PPD and prolonged peak latency with a degraded performance due to additive background noise [18].

**Figure 1:**
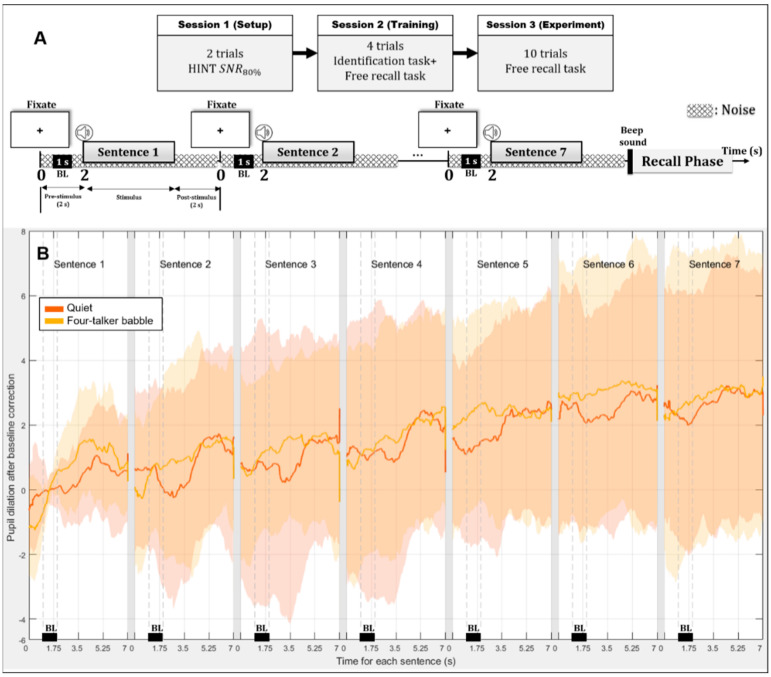
Outline of experimental design. (**A**) Three sessions including tasks given to each participant and encoding and recall phases used in the present study; (**B**) time course of averaged pupil dilation during the encoding phase of a seven-sentence list across participants for each listening condition (quiet or four-talker babble noise). Sentence baselines were corrected with respect to the initial-sentence baseline (sentence 1), and peak pupil dilation values were corrected using the sentence’s baseline. Pupil dilation curves: Orange, in the quiet condition; yellow, in the noise condition. Orange and yellow shaded areas indicate standard deviations. “0” represents the onset of noise used to calculate peak pupil latency. BL, 1-s baseline period during the 2-s pre-stimulus (noise-only) period.

Baselines were typically used for scaling purposes and previous studies have paid little attention to baselines or its interaction with task-evoked responses [21,22]. Baseline as an independent analysis parameter is known to closely relate to sustained processing while performing a cognitive task, suggested by Siegle et al. [23], who reported an increase in baseline with increasing task demands. However, we note that our study recorded pupil positions and diameters for each stimulus presentation to calculate mean effects of the abovementioned variables in a given trial (see details in Figure 1), instead of the previously used trial-by-trial measure that has been used in speech-recognition studies. In recall-based experiments, each trial consists of a series of successively presented stimuli. Therefore, we assumed that analyzing the pupil data while maintaining the order of presentation should be considered for the current study design and used a different approach to baseline correction where the baseline of the initial stimulus on a list was the basis of all the post-hoc analysis (which will be explain later in more detail).

We aimed to assess feasibility of pupillometry in normal-hearing listeners simultaneously performing a free-recall task modified from [11,13]. Experiment 1 examined recall performance of spoken words in stationary noise and its correlation with RS scores. Experiment 2 measured recall performance of spoken words in both quiet and four-talker babble noise conditions and assessed its correlation with RS scores. The effect of noise on pupil responses was also investigated by analyzing the pupil responses during the encoding phase. Our hypotheses were as follows: (1) Listeners with higher WMC might outperform those with lower WMC at free recall of spoken words regardless of noise interference; (2) encoding of speech in background noise, although speech is easily audible, might impair memory performance of the listeners irrespective of the serial position effect, even though they have apparent normal hearing; (3) RS might be more strongly correlated with memory performance of speech in background noise than in quite; and (4) how pupil responses, indicated by sentence baselines, PPDs and latencies to peak dilates relative to the baseline might be influenced by the noise interference and/or the order of stimulus presentation regarding more effortful listening or memory demand.

## 2. Materials and Methods

### 2.1. Participants

Native Korean speakers with normal hearing were enrolled: 25 participants in the first experiment (mean age 28 years, range 19–44 years) and 34 participants in the second experiment (mean age 28.4 years, range 20–39 years); some of the participants participated in both experiments (Table 1). Participants with no prior history of ear infection or surgery, diabetes mellitus, or middle-ear dysfunction were included; all provided written informed consent. They were paid for their participation and asked to arrive for testing without applying any eye make-up (i.e., eyeliner or mascara). Normal hearing was defined as pure-tone air-conduction thresholds ≤20 dB HL at 0.5, 1, 2, and 4 kHz. The Korean version of the Montreal Cognitive Assessment (MoCA-K) was used to ensure normal cognitive functioning with a cut-off score of ≥23 out of 30 points [24]. The MoCA-K is a rapid and sensitive test for detecting mild cognitive impairment and requires 10 to 12 min to complete. It includes 30 questions that evaluate trail making, cube copying, clock drawing, naming, immediate memory, attention, language, abstraction, delayed recall, and orientation. Participants’ levels of education ranged from high school graduates to postgraduate degree holders.

### 2.2. Equipment and Test Materials

Experiments were performed inside a double-walled, sound-attenuating booth. All auditory stimuli were preprocessed using Matlab version 9.4 and were presented via a loudspeaker (8040 BPM; Genelec, Iisalmi, Finland) and a high-quality 24-bit external PC soundcard (MAYA 44 USB + Soundcard, ESI, Leonberg, Germany) with SoundMexPro (Hoertech, Oldenburg, Germany). For stimuli, we selected 98 sentences from the Korean Hearing in Noise Test (KHINT) [25], originally containing 25 lists of 10 sentences suitable for elementary school students (grades 4–6), while maintaining 70–80% of sentence recognition; target words were placed at the beginning of each sentence. These stimuli were selected as described in Ng et al. [13] and Lunner et al. [11] after excluding rare or duplicate words within a trial, considering word frequency, keyword length, syllable number, and word difficulty, and ensuring that word difficulty was evenly distributed across the lists. Then, each list was phonetically-balanced and we selected more frequent words, but excluded those sentences where the initial word starts with pronouns or duplicate words. The final set of stimulus materials consisted of 14 seven-sentence lists with four practice lists. There was an average of 1.94 syllables in the target words. To evaluate serial position effects, as described by Ng et al. [13], sentences in each list were sub-grouped as follows: the first and second sentences to the primacy, third to fifth sentences to the asymptote (middle in the list), and sixth and seventh sentences to the recency position. The standardized KHINT sentences were recorded by a professional male voice actor. However, we modified the recordings to obtain equal loudness and match the average frequency spectra of the selected sentences for the identification and free-recall tasks. Four-talker babble noise was generated from four native Korean speakers (two males and two females) who read four different paragraphs of text. Then the babble noise was filtered through the long-term average spectrum of the chosen sentences for the identification and free-recall tasks. The KHINT noise was used as stationary noise in Experiment 1. A wearable eye-tracking headset (Pupil Core; Pupil Labs, Berlin, Germany) with 200-Hz binocular cameras was positioned in front of the participant’s eyes to record changes in pupil diameter.

### 2.3. Procedure

Auditory stimuli were presented via a single loudspeaker, and a free-recall task modified from [11,13] was used in both experiments. Participants sat 1 m in front of the loudspeaker and listened to the target speech fixed at 65 dB SPL with or without noise. During listening to a list of seven sentences, participants were instructed to stare at a dot, positioned at a distance of 1 m, immediately above the speaker to minimize the effect of the light reflex on the pupil response. 

All enrolled participants completed three sessions per experiment, which required approximately 1.5 h on the day of their visit (Figure 1). The SNRs used in the two noise conditions (stationary noise in Experiment 1; four-talker babble noise in Experiment 2) were calculated during the KHINT and four training lists depending on the repetition performance only. Session 1 began with KHINT under a noise condition to obtain individual speech reception thresholds at 80% of correct performance (number of target words correctly repeated) using a 4-up-1-down adaptive procedure [25]. In Session 2, during training with four practice lists, participants completed both repetition and free-recall tasks. The repetition task was to repeat the initial word of the sentence after each sentence, while the participants were trying to remember as many words as possible to recall them in any order after each list of seven sentences. In this training, the initial SNR threshold was tuned to reach the equivalent of 95% of correct repetition performance, although listeners were instructed to complete both tasks, repetition and free recall. The noise was decreased until the participant correctly repeated six or seven target words (in 1-dB steps if they correctly repeated 4 or 5 words and in 2-dB steps if they correctly repeated 0 to 3 words), as described in [11]. The recall phase began with the presentation of a 0.2-s beep sound, and participants were prompted to recall all sentence-initial words. The examiner recorded the number of words correctly repeated and recalled as a function of serial position.

Session 3 included a free-recall task concurrently with pupil diameter recording, and participants were asked not to give any verbal response before the beep in order to prevent rehearsal of to-be-remembered items that might potentially influence subsequent recall performance. To avoid the effects of fatigue on memory performance [26], breaks were allowed between lists, if necessary; for instance, a brief break of 2 min was given during the KHINT and a brief break of 3 min was given during the free-recall task.

### 2.4. Reading Span Test

The RS test included 12 lists, each consisting of 100 sentences [27]. Participants were instructed to report the last word of each sentence, in order, immediately after a set of sentences was sequentially displayed on a screen using Matlab software. The examiner simultaneously recorded their responses. This RS version had five trials per level, from Level 2 to Level 6, such that the number of sentences in a set corresponded to the level; for instance, Level 2 contained five sets of two-sentence trials and Level 3 contained five sets of three-sentence trials. Individual RS was evaluated with a maximum of six points using a scoring method described below. If a participant correctly recalled at least three of the five sets in a level, the participant received a score equivalent to that level and moved to a higher level. A participant who recalled two of the five sets at a particular level was given one-half point and the test was stopped after that level. Finally, 0 points were given to a participant who recalled one or zero sets at a particular level. The median score of participants was 4.0 points in Experiment 1 and 4.75 points in Experiment 2. In Experiment 1, participants who scored less than 4.0 were categorized as low-RS (*N* = 9) and the remaining participants were categorized as high-RS (*N* = 16). In Experiment 2, participants who scored 4.75 or less were categorized as low-RS and the remaining participants were categorized as high-RS (*N* = 17 in each group *N* = 17).

### 2.5. Pupil Diameter Recording and Data Analysis

An adequate amount of rest (~3 min) was given between lists and sessions. Prior to data collection, the light intensity was individually adjusted to the pupil-size midpoint, from dim (~30 lux) to bright (~230 lux). The examiner was cautious about excessive blinks during the encoding of sentences and regularly reminded all listeners to maintain their gaze fixed on the black dot. Pupil diameters were recorded in millimeters using three-dimensional pupil detection software provided by Pupil Labs (Germany) and Matlab software generating annotations during the recording (each list of seven sentences). The start of noise, a sentence, and a recall period. All pupil data were processed in accordance with the proposed four-step method in the pupillometry data-processing guidelines [28] using Matlab. First, we cut the recording into sentences (or traces) by setting the annotation times (the start of noise before each sentence) to zero (Figure 1). Next, based on the time range (onset and offset of noise) set by an examiner, the correct portion of pupil data was selected. Then, the traces were pre-processed to remove samples with blink artifacts or dilation speed outliers using median absolute deviation method [28]. Pupil diameter values greater or lower than the median ±2.5 times the standard deviation of the remaining data were defined as blink artifacts. A miss rate of 30% was applied for blink detection and data selection. After removing blink artifacts, the selected traces were passed through a moving average and the blinks were interpolated. Finally, the baseline of the first sentence was computed with the time range parameter and was used to correct all sentence baselines, so by design the baseline of each first sentence in each list was set to 0. 

Divisive baseline correction (proportional change from baseline) was applied to the pre-processed data for PPDs of each sentence. However, because this study used consecutive non-independent trials, a different way of correction was used: Sentence baselines were corrected with respect to the first initial baseline, which was set to 0 in the first analysis after acquisition, then the PPD values were corrected from the sentence’s baseline. The baseline was calculated as the mean pupil diameter during the final 1 s of the pre-stimulus period and was measured before each sentence. The change in pupil size calculated relative to the pre-stimulus baseline was defined as pupil dilation, the highest amplitude of pupil dilation after sentence onset was defined as PPD, and the time taken to reach PPD was defined as peak latency. Therefore, three dependent variables (baseline, PPD, and peak latency) were computed in the present study. Valid pupil data were collected from 34 listeners whose pupil traces met inclusion criteria for analyses. 

### 2.6. Statistical Analyses

The percentage of correctly recalled words in Experiment 1 (*N* = 25) was analyzed by two-way nonparametric repeated measures analysis of variance (RMANOVA) with a within-subjects factor (serial position) and a between-subjects factor (RS group) because the recall score was a discrete variable rather than a continuous one that follows a normal distribution. To control Type 1 error due to multiple comparisons and/or hypothesis testing, post hoc analyses with Bonferroni correction were used and α was set to 0.0167 for these contextual comparisons. The R package nparLD was used for nonparametric analyses [29,30]. The effects of noise were not examined in Experiment 1 because stationary noise was used. A *p* value < 0.05 was considered significant. Using SAS MACRO run_npar (version 8.0; http://www.ams.med.uni-goettingen.de/makros/run_npar.html (accessed on 23 August 2000)), the percentage of correctly recalled words in Experiment 2 (*N* = 34) was analyzed by three-way nonparametric RMANOVA with a within-subjects factor (noise) and the above two factors; then, for significant variables, post hoc analysis was performed with the Bonferroni-corrected α value of 0.0167 (=0.05/3). However, gender difference in the recall performance was not statistically analyzed because it was not of primary interest.

Pupil diameter data in Experiment 2 were analyzed by linear mixed models with two fixed effects for noise (quiet or four-talker babble noise) and stimulus presentation order, an interaction between noise and stimulus presentation order, and a random effect for subject because the linear mixed models allow to handle values missing due to a large number of blinks. Pupil diameter data were collected from both right and left eyes and the dependent variables were sentence baselines, PPDs, and latencies to peak. The 95% confidence intervals were calculated by using the standard error obtained from the estimated variance-covariance matrix of parameter estimates. Statistical comparisons of pupil data between the two RS groups were not feasible because of the individually adjusted luminance.

## 3. Results

### 3.1. Free Recall of Spoken Words in Stationary Noise

**Figure 2:**
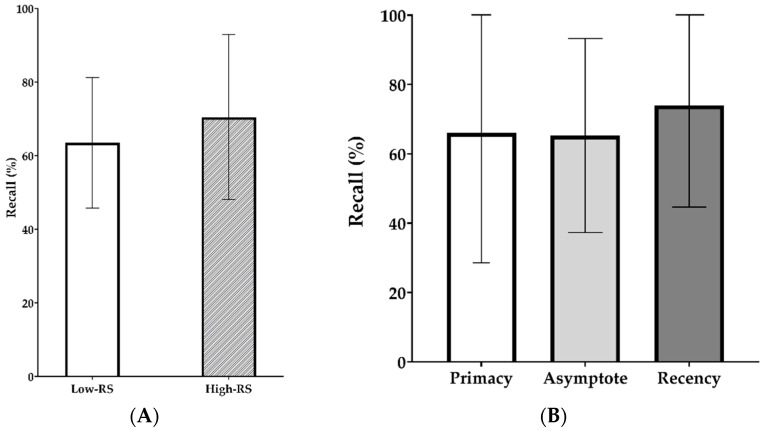
Results of Experiment 1. Mean recall percentages in the stationary noise condition (**A**) as a function of reading span (RS) group (low- and high-RS groups); (**B**) as a function of serial position (primacy, asymptote, and recency).

Mean recall percentages for the stationary noise condition are shown as a function of RS group (low or high) in Figure 2A and as a function of serial position (primacy, asymptote, and recency) in Figure 2B. As expected, the high-RS group tended to perform better than the low-RS group in the free-recall task in the presence of stationary noise; however, the nonparametric RMANOVA revealed no significant differences between the two RS groups. Recall performance was higher for recency position items than for items in other positions, but there was no significant serial position effect nor interaction between RS group and serial position.

### 3.2. Free Recall of Spoken Words in Quiet Condition versus Four-Talker Babble Noise

**Figure 3:**
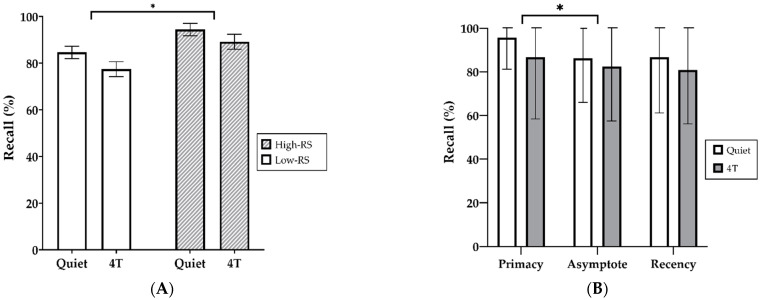
Results of Experiment 2. Mean recall percentages in the modified recall task of spoken words in quiet and four-talker babble (**A**) as a function of reading span (RS) group (low- and high-RS groups); (**B**) as a function of serial position (primacy, asymptote, and recency), * *p* < 0.0167.

Mean recall percentages for the quiet and four-talker babble conditions are shown as a function of RS group (low or high) in Figure 3A and as a function of serial position (primacy, asymptote, and recency) in Figure 3B. We found lower recall performance in the four-talker babble noise condition than in the quiet condition, albeit the difference was not significant; there was no significant fixed effect of noise nor its interaction with serial position. These two insignificant variables were excluded from further analysis, and the nonparametric RMANOVA revealed significant fixed effects of RS group (*p* = 0.0018) and serial position (*p* = 0.0269) on the free-recall performance. This indicates that, regardless of the noise type, the high-RS group performed better at the free-recall task than did the low-RS group. In addition, after correction for the group variable (RS group), post hoc analysis revealed significantly higher performance for the primacy items than for the asymptote items (*p* = 0.0079). 

We also tested each of the three variables of interest (RS group, serial position, noise) individually after correction for the other two variables, and nonparametric RMANOVA revealed that two of them (serial position and RS group) remained significant: *p* = 0.0323 and *p* = 0.0016, respectively. 

### 3.3. Correlation between Free Recall and RS Scores

**Figure 4:**
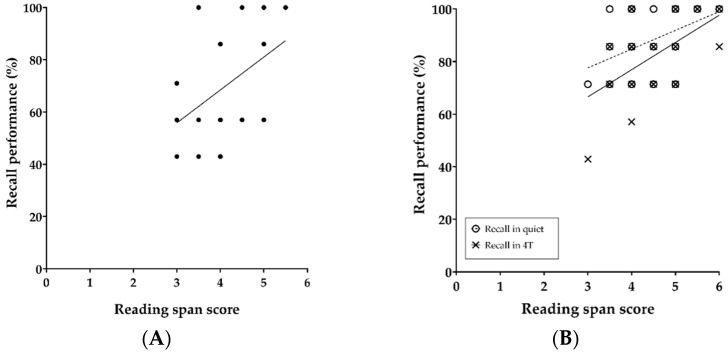
Scatterplots of recall performance versus reading span (RS) scores. (**A**) in stationary noise (*N* = 25); (**B**) in the quiet condition (dashed line) and four-talker babble noise (solid line) (*N* = 34).

Correlations between free recall and RS scores are shown in Figure 4. RS was moderately positively correlated with recall performance in the stationary noise condition (r = 0.454, *N* = 25, *p* = 0.0225) (Figure 4A). RS was moderately positively correlated with recall performance in the quiet condition (r = 0.424, *N* = 34, *p* = 0.0125) and more strongly in the presence of four-talker babble noise (r = 0.512, *N* = 34, *p* = 0.002) (Figure 4B).

### 3.4. Sentence Baselines Relative to the Initial-Sentence Baseline during Encoding

**Figure 5:**
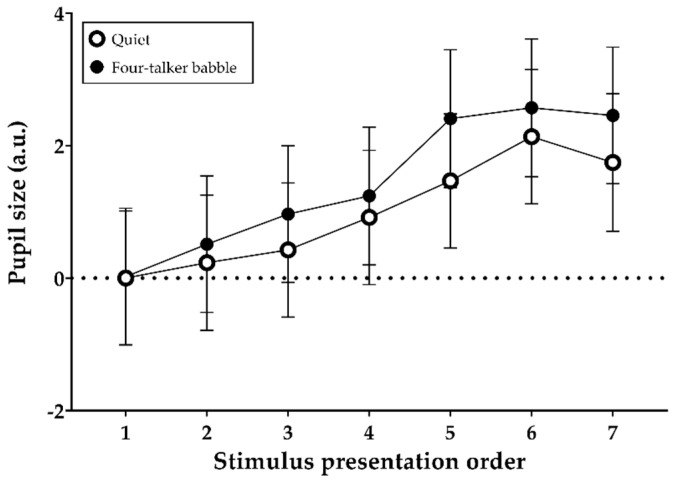
Mean and 95% confidence intervals of sentence-baseline values relative to the initial stimulus baseline in quiet and four-talker babble conditions as a function of stimulus presentation order.

Figure 5 shows the mean (and 95% confidence intervals) sentence-baseline values in the quiet and four-talker babble noise conditions during the encoding of auditory information into memory. Both fixed effects of noise (F (1, 705) = 6.49, *p* = 0.011) and stimulus presentation order (F (6, 701) = 14.87, *p* < 0.001) on the baselines were significant. Relative to the initial sentence baseline, baseline sequentially increased with each sentence in line with the increasing memory load (number of items to be remembered). Moreover, the baseline of each sentence in the quiet condition was significantly lower than that in the noise condition regardless of presentation order. The interaction between noise and stimulus presentation order was not statistically significant. 

### 3.5. Peak Pupil Dilations during Stimulus Presentation

**Figure 6:**
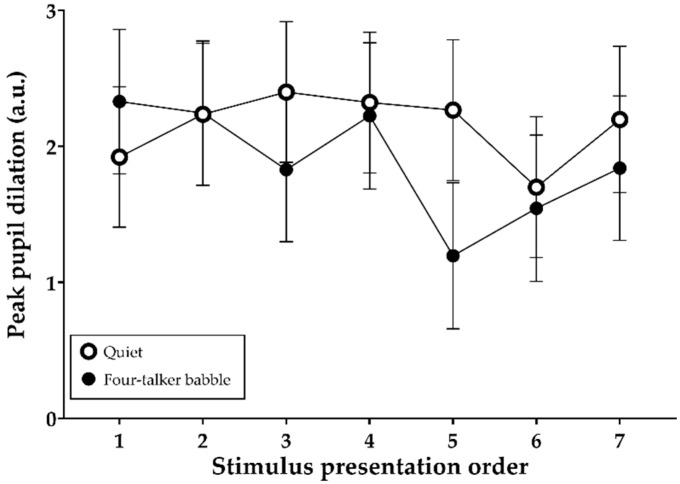
Mean and 95% confidence intervals of peak pupil dilations in the quiet and four-talker babble conditions as a function of stimulus presentation order.

Figure 6 shows the mean (and 95% confidence intervals) PPD values in the absence or presence of four-talker babble noise during the encoding of auditory information into memory. Both fixed effects of noise (F (1, 709) = 5.18, *p* = 0.023) and stimulus presentation order (F (6, 701) = 2.76, *p* = 0.012) on the PPDs were significant. Except for the PPD value of the initial sentence, the overall sentence-PPDs were larger in the quiet condition than in the noise condition. The interaction between these two variables was also significant (F (6, 701) = 2.45, *p* = 0.024). 

### 3.6. Peak Pupil Latencies during Stimulus Presentation

**Figure 7:**
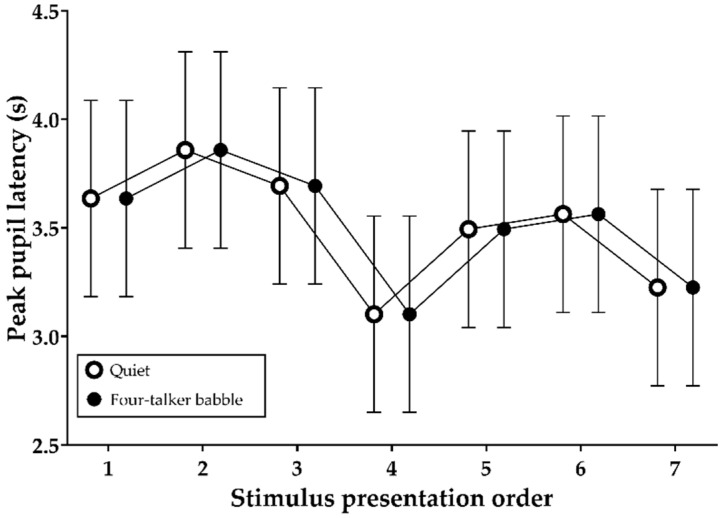
Mean and 95% confidence intervals of peak pupil latencies in the quiet and four-talker babble conditions as a function of stimulus presentation order.

Figure 7 shows the mean (and 95% confidence intervals) peak pupil latency values during the encoding of auditory information into memory in the absence or presence of four-talker babble noise. A fixed effect of stimulus presentation order was significant (F (6, 770) = 4.01, *p* = 0.001). The latencies were identical between the quiet and four-talker babble noise conditions, and the latency was shortest after the 4th sentence and longest after the 2nd sentence.

## 4. Discussion

In this study, we explored the possibility of elucidating the neural basis of cognitive processing in hearing by pupillometry concurrent with a free-recall task. Noise did not seem to significantly affect the free-recall performance of normal-hearing adults, but the other variables (serial position and RS group) had a significant effect on the performance. Overall, normal-hearing listeners with higher WMC, indexed by RS test, performed better than did listeners with lower WMC (see Figure 2A and Figure 3A). Significantly poorer recall was seen in noise than in the quiet condition, showing that noise has a negative impact on memory performance of normal-hearing listeners, even though the speech was fairly intelligible. These findings are consistent with previous studies, which imply that hearing acuity and cognitive ability strongly aid understanding of degraded speech [3,5,6,31]. Furthermore, RS positively correlated with free-recall scores measured in the quiet condition and in two types of noise conditions. Last, in four-talker babble noise, which required more effortful listening, significantly larger sentence baselines but smaller PPDs were observed. 

As to hypothesis 1, our study demonstrated that the high-RS group outperformed the low-RS group at free recall of spoken words in the quiet and four-talker babble noise conditions (Figure 3A), albeit the difference was not significant in the stationary noise condition (Figure 2A). However, because of an insufficient number of participants in Experiment 1, this needs to be confirmed in a further study involving a larger population. The hypothesis that RS scores would predict listeners’ free-recall accuracy of spoken words has been previously supported by testing hearing-impaired listeners in the absence or presence of noise reduction [11,13].

The free-recall performance of normal-hearing adults tended to be degraded when the target words were heard in noise (Figure 3), but this effect was not statistically significant. Different types of noise or listening conditions are needed to prove hypothesis 2 about the impact of noise interference or noise types on free recall.

Free recall in normal-hearing adults followed a pattern similar to that in previous studies that tested hearing-impaired listeners [11,13]; primacy effect was present in recall performance when the listeners heard the speech in Experiment 2 regardless of the noise interference (quiet versus four talker babble condition). However, the recency effect in the stationary noise condition was not significant. However, there was no significant effect of serial position or interaction of serial position with other factors on free recall when speech was degraded by stationary background noise, which suggests that speech babble was a more effective masker than stationary noise in a recall-based task. Participants felt easier to recall words in the primacy or recency position than those in the asymptote position regardless of the noise conditions. In contrast to the results from similar studies [11,13], primacy effect was stronger than recency effect in the quiet condition (see Figure 3B); however, a direct comparison could not be made with those studies due to differences in noise reduction, statistical methods, study population, and instruction.

RS was positively correlated with free-recall performances under the quiet condition and two different types of noise conditions (see Figure 4). In particular, RS strongly correlated with recall performance in competing speech, in contrast to the previously reported weak correlation between RS and the performance in four-talker babble noise [13]. This finding may imply that recall scores in four-talker babble noise can be a more precise predictor of RS scores, and this supports the feasibility of using the modified task applied in this study as an indicator of cognitive spare capacity. In this regard, we conclude that hypothesis 3 is partially validated. It is plausible that altered recall instruction is more advantageous for obtaining reliable behavioral results from fluent Korean speakers. In addition, the nature of the background noise and target speech seems to have influenced the association between the performances of these two cognitive assessments (RS and recall of auditory information). Although, different types of stimuli were used. The instruction to remember the final word of each sentence might be less suitable for native Korean speakers, who are unfamiliar with subject-prominent languages and subject-verb-object sentence structure. Distinct from the languages of other published SWIR versions (i.e., Swedish or Danish), the Korean language uses a subject-object-verb structure and high-context cultural characteristics [24]. A widely shared assumption in Korea’s high-context culture is that not every word in a sentence is needed to convey the full meaning of that sentence. In addition, pronouns are frequently omitted. Indeed, subjects and objects used at the beginning of sentences are not likely to be specified. This is also a common feature in topic-prominent languages, such as Korean. As a result, in this study, we altered the existing task instruction to consider the different type of sentence structure in which participants memorized the first words of a sentence. The dual-task paradigm described by Sarampalis et al. [12] also required free recall of final words to investigate whether memory performance differed depending on different amounts of contextual information. Sarampalis et al. [12] demonstrated that in normal-hearing adults the primacy effect was conditional on the SNR level of the stimuli and noise-reduction processing. 

With regard to hypothesis 4, we found significantly larger sentence baselines but smaller PPDs as a result of speech mixed with interfering noise and/or the order of stimulus presentation (Figure 5 and Figure 6). Apparently, sentence baselines relative to the initial stimulus baseline increased linearly with memory load (number of words to be remembered). In addition, the overall baseline values were smaller in the noisy listening condition than in the quiet condition. Noise and stimulus presentation order seemed to affect pupil responses of normal-hearing listeners engaged in a free-recall task. However, we initially noted that our pupil-response analysis was based on a single recording per test condition, which can be a limitation of our study according to a recently published practice guideline for pupillometry measure [26], which highly recommends averaging across multiple trials to minimize task-irrelevant changes in pupil response. Unlike the pattern found in sentence baselines, significantly lower PPDs were found in the noisy listening environment. However, these results were contrary to our expectations that speech encoding in noise would be associated with elevated PPD and longer peak latency because listeners would feel that more effort was required. Our initial hypothesis, supported by the work of Zekveld et al. [18], was that speech processing with addition of noise might increase PPD and peak latency. The unexpected pupil response that we observed needs to be confirmed in a further study involving a sufficient number of pupillometry trials. Furthermore, in terms of the significant reductions in PPD, we did not expect to observe disengagement by listeners [32,33] because the speech was presented at nearly 90% intelligibility. Prior studies of pupil responses to degraded speech have reported increasing PPD at up to 50% correct sentence recognition. Another plausible explanation is that some unidentified influence of the recall task might have been present in the recording because pupillometric recording is reportedly affected by several other factors, such as age, hearing status, room lighting, anxiety, attention, and fatigue [26,34,35,36]. At this preliminary stage of our pupillometry research, the procedure requires additional design optimization because little is known about pupil responses during encoding of spoken words. Further research is needed to clarify the effects of recall task on pupil response using repeated trials in a single-task block.

## 5. Conclusions

The analysis method of pupil dilation, used in this study, is likely to provide a more thorough understanding of how listeners respond to a later recall task in comparison with previously used methods. Further studies are needed to confirm the applicability of our method in people with impaired hearing using multiple repetitions to estimate the allocation of relevant cognitive resources.

## Figures and Tables

**Table 1:** Demographics of all participants.

Variable	First Experiment (*N* = 25)	Second Experiment (*N* = 34)
	Mean ± SD	Mean ± SD
Age	28 ± 7.6	28.4 ± 5.5
Sex		
Female	22 (88%)	18 (53%)
Male	3 (12%)	16 (47%)
Hearing threshold (dB HL)	6 ± 3.3	3 ± 4.3
RS score	4 ± 0.7	4 ± 0.1
MoCA-K		
Total score	28 ± 0.3	29 ± 1.7
Delayed recall	3.8 ± 1.14	4 ± 1.0

RS, reading span; hearing threshold as pure-tone average of four frequencies (0.5, 1, 2, and 4 kHz); MoCA-K, the Korean version of the Montreal Cognitive Assessment.

## Data Availability

The data presented in this study are available on request from the corresponding author. The data are not publicly available due to participant privacy.
